# Evaluation of brachial artery diameter and flow-mediated vasodilatation as tools to predict cardiovascular events in type 2 diabetes patients

**DOI:** 10.1186/1758-5996-7-S1-A40

**Published:** 2015-11-11

**Authors:** Lorenzo Catucci Boza, Cristine Kasmirscki, Sandro Antunes da Silva, Maira Zoldan, Thiago Barth Bertotto, Tiago Tonelli, Alice Hoefel Nunes, Luis Eduardo Paim Rohde, Jorge Luiz Gross, Mirela Jobim de Azevedo, Themis Zelmanovitz

**Affiliations:** 1 Universidade Federal do Rio Grande do Sul, Porto Alegre, Brazil

## Background

It has been suggested that brachial artery flow-mediated dilatation (FMD) evaluated by Doppler ultrasound, as a measurement of endothelial function, may predict cardiovascular events in healthy people. However, the data about this test in diabetic patients is scarce.

## Objective

To evaluate the performance of brachial artery basal diameter and FMD in predicting cardiovascular risk determined by validated scores (reference standards).

## Materials and methods

In this diagnostic test study, the type 2 diabetic patients were submitted to clinical and laboratory evaluation. Endothelial function was evaluated with Doppler ultrasound of the brachial artery, measuring the basal diameter and the FMD after ischemia in the forearm. ROC curves were constructed and two scores were used as reference standards to assess the risk of having a cardiovascular event over 10 yrs.: UKPDS risk engine (<10%=low risk and ≥ 10%=high risk) and ASCVD (<7.5%=low risk and ≥ 7.5%=high risk). The cutoff points of basal diameter and FMD were determined based on the equilibrium between sensitivity (S) and specificity (E).

## Results

The study included 154 patients with type 2 diabetes and clinically free of cardiovascular disease (59.7% female, mean age 63±9 yrs., diabetes duration 16 (9– 21) yrs.). When UKPDS risk engine was used as reference standard, the area under the curve (AUC) was 0,604±0.063 (CI=0,515-0,698; P=0.083) for FMD, with a S=47.2% and a E=75% for the cutoff point ≤ 5.23%. For basal diameter, the AUC was 0,648±0.056 (CI=0,554-0,734; P=0.019), with a S=76.4% and a E=50% for the cutoff point >0,306. When ASCVD score was applied, the AUC was 0,628±0.064 (CI=0,538-0,712; P=0.045) for FMD, with a S=77.2% and a E=57.7% for the cutoff point ≤ 8,17%. For basal diameter, the AUC was 0.7±0.052 (CI=0,613-0,778; P=0.002), with a S=86.1% and a E=50% for the cutoff point >0.302.

## Conclusion

Both FMD and basal diameter of brachial artery evaluated by Doppler ultrasound presented a low to moderate accuracy to predict cardiovascular risk, determined by UKPDS risk engine and ASCVD scores in patients with type 2 diabetes. It is probably due to an overlap of the values of these tests between the high and low risk patients. Longitudinal studies evaluating cardiovascular outcomes in these patients are needed to clarify these findings.

